# Selenium Alleviates Cerebral Ischemia/Reperfusion Injury by Regulating Oxidative Stress, Mitochondrial Fusion and Ferroptosis

**DOI:** 10.1007/s11064-022-03643-8

**Published:** 2022-06-20

**Authors:** Yuanyuan Shi, Lijian Han, Xianxian Zhang, Lili Xie, Pinglei Pan, Fei Chen

**Affiliations:** 1 Department of Central Laboratory, The Yancheng School of Clinical Medicine of Nanjing Medical University (Yancheng Third People’s Hospital), Yancheng, 224008 Jiangsu China; 2 Department of Neurology, The Yancheng School of Clinical Medicine of Nanjing Medical University (Yancheng Third People’s Hospital), Yancheng, 224008 Jiangsu China

**Keywords:** Cerebral ischemia/reperfusion, Selenium, Oxidative stress, Ferroptosis, Mfn1, Mitochondrial fusion

## Abstract

To clarify the potential role of selenium (Se) on cerebral ischemia/reperfusion (I/R) injury, we utilized mouse middle cerebral artery occlusion (MCAO) followed by reperfusion as an animal model and oxygen–glucose deprivation and reoxygenation (OGD/R) to treat N2a cells as a cell model, respectively. MCAO model was established in mice and then divided into different groups with or without Se treatment. TTC staining was used to observe whether the cerebral I/R modeling was successful, and the apoptosis level was determined by TUNEL staining. The expression of GPx-4 and p22phox was assessed by western blot. In vitro experiments, the OGD/R induced oxidative stress in N2a cells was assessed by levels of GSH/GSSG, malondialdehyde, superoxide dismutase and iron content, respectively. QRT-PCR was used to detect the mRNA levels of Cox-2, Fth1, Mfn1 and mtDNA in N2a cells. JC-1 staining and flow cytometry was performed to detect the mitochondrial membrane potential. Se treatment alleviated cerebral I/R injury and improved the survival rate of mice. Additionally, Se treatment apparently attenuated oxidative stress and inhibited iron accumulation in MCAO model mice and OGD/R model of N2a cells. In terms of its mechanism, Se could up-regulate Mfn1 expression to alleviate oxidative stress and ferroptosis by promoting mitochondrial fusion in vivo and vitro. These findings suggest that Se may have great potential in alleviating cerebral I/R injury.

## Introduction

Ischemic cerebral vascular disease has the characteristics of high incidence rate, disability rate and mortality, which can lead to different degrees of neurological disorders and seriously threaten public health [1, 2]. Brain injury caused by ischemia/reperfusion (I/R) refers to the phenomenon that the brain experiences permanent or temporary ischemia, followed by the sudden recovery of blood supply [3]. I/R injury lead to not only the failure of brain function recovery, but also more serious neuronal dysfunction. Therefore, alleviating I/R brain injury is an urgent problem to be solved in the treatment of ischemic cerebrovascular disease.

Accumulating evidence has revealed that infiltration of inflammatory cells and overexpression of inflammatory factors after cerebral I/R are able to trigger inflammatory response and aggravate ischemic injury [4]. Brain injury caused by I/R is often irreversible, accompanied by neuronal damage and death, involving a variety of pathophysiological processes, such as oxidative stress, calcium overload, toxic injury, and so on [5]. Among them, oxidative stress interacts with inflammation during cerebral I/R injury, which is of great significance in regulating the pathological process [4, 6]. As we all know, mitochondria are generally regarded as the energy factory of cells because of their ability to produce ATP. Besides, as an important energy supply organelle, mitochondria are in a dynamic balance of continuous movement, fusion and division. The disorder of mitochondrial dynamic balance can lead to a variety of diseases, mainly nervous system diseases, affecting neuronal development, plasticity and functional defects [7]. Based on the above, we recognize that oxidative stress and mitochondrial function are of great significance in the regulation of cerebral I/R injury.

Selenium (Se) is necessary for a variety of metabolic processes, including thyroid hormone metabolism, protection against oxidative stress, and immunity functions, which is an essential trace element for human and animal health [8]. Se also is the activator of glutathione peroxidase (GPX), which regulates the antioxidant mechanism to prevent oxidative damage [9].

Recent studies demonstrated that secondary cell death after intracerebral hemorrhage might be driven by ferroptosis rather than the random destruction of macromolecules activated by iron catalytic oxidants [10–12]. Ferroptosis is a kind of cell death triggered by iron-dependent lipid peroxidation [13], which can be induced by excessive secretion of lipid peroxidase or selectively blocked by lipid peroxidation inhibitors [14]. In addition to antioxidant activity, Se has been proved to have a potential role in ferroptosis. The researchers suggested that even under nutrient rich conditions, appropriate Se supplementation unexpectedly drove adaptive transcription to combat ferroptosis, thereby protecting neurons from I/R injury [15]. More importantly, cysteine deficiency will cause hyperpolarization of mitochondrial membrane potential (MMP) and excessive accumulation of lipid peroxides, and finally the damaged mitochondria promote the occurrence of iron sagging [16, 17].

Herein, combined with previous studies, we aimed to clarify the therapeutic effect of Se on cerebral I/R injury. We hypothesized that Se can upregulate the expression of Mfn1, thus promoting mitochondrial fusion. Moreover, Se could reduce the level of oxidative stress, so as to weaken the cell ferroptosis after cerebral I/R injury.

## Methods

### Animals

All mice were purchased from Laboratory Animal Department of Chinese Academy of Medical Sciences (Beijing, China), and housed in pathogen-free cages under conditions controlled for temperature 22 ± 1 °C, humidity 50% ± 10%, a cycle of 12 h/12 h light/dark, and freely with a standard rodent diet and water. All procedures were carried out in accordance with the National Institutes of Health Guide for the Care and Use of Laboratory Animals. Randomization and blinding were used in animal experiments.

### Stereotaxic Injection

After anesthetized, each mouse was bound to a stereotaxic apparatus. Using RNAiMAX transfection reagent (ThermoFisher, Waltham, MA, USA), 5 μl lentivirus of sh-NC or sh-Mfn1 (10^9^ units/Ml, Shanghai GenePharma) was mixed and then added into the lateral ventricle (0.2 mm behind the bregma, 1.0 mm outside the center line, and 1.5 mm below the brain surface). Twenty-four hours later, the mice suffered from middle cerebral artery occlusion (MCAO) treatment.

### MCAO Treatment

The mice were subjected to MCAO operations according to the previous description [18]. Briefly, after the mice were anesthetized with 3% isoflurane, the common carotid artery (CCA), external carotid artery (ECA), and internal carotid artery (ICA) of mice were exposed under a microscope, respectively. The nylon wire from CCA were inserted into ICA, and then transferred to the starting point of ECA until it blocked the starting point of middle cerebral artery (MCA). After 1 h of occlusion, the nylon suture was removed for reperfusion for 24 h.

For the Se treatment, mice were administered by intraperitoneal injection of 0.5 mg/kg/day of selenite sodium (S5261, Sigma) for 7d before the MCAO operation [19].

### 2,3,5-triphenyltetrazolium Chloride (TTC) Staining

To determine the volume of cerebral infarct, TTC staining assay was performed. In brief, the mouse brains were collected and cut into 2-mm thick sections. Then, the sections were stained with TTC solution (2%, LJ0508B1009J, BIO BASICINC) for 30 min at 37 °C. The normal brain tissues were colored as red but the infarcted brain tissues remained unstained as white. The infarcted areas were analyzed by Photoshop software.

### TUNEL Staining Assay

The TUNEL assay was carried out to quantify apoptotic level. Briefly, tissue slides were washed and fixed at room temperature, then permeabilized with inproteinase K solution for 10 min. TdT enzyme andfluorescein-12-dUTP were added onto the tissue slides, which then incubated for 2 h at 37 °C. Following incubation, the tissue section were treated with stopping buffer for 5 min, and then washed with PBS. Finally, TUNEL-positive cells were visualized using a fluorescence microscopy.

### Cell Culture, Transfection and Treatment

N2a neuroblastoma cells were obtained from Shanghai Institute of Cell Biology, Chinese Academy of Sciences (Shanghai, China). Cells were kept in DMEM containing 10% fetal bovine serum (HyClone, Logan, UT, USA) and 0.1% penicillin/streptomycin at 37 °C in a humidified incubator with 5% CO_2_ atmosphere. For the treatment, cells were incubated with the addition of selenite sodium (10 μM) in the specific group. And to differential expressed Mfn1, the lentiviral vector pLKO-TRC-puro containing shRNA against Mfn1 (shMfn1: 5′-TGCAATGCTGTGGGATAAAGT-3′) or non-targeting control (shNC: 5′-TGCGCTAGGCCTCGGTTGC-3′) were transfected into N2a cells using the Lipofectamine™ 2000 kit (Invitrogen).

### Oxygen and Glucose Deprivation/Reoxygenation (OGD/R) in N2a Cells

To establish the OGD/R mode, N2a cells were subjected to OGD by cultured with deoxygenated glucose-free Hanks’ Balanced Salt Solution (Beyotime Institute of Biotechnology, Jiangsu, China) and incubated in a hypoxic chamber including 95% N_2_ and 5% CO_2_ at 37 °C for 2 h. After that, cells were incubated with normal culture medium at 37 °C for 24 h under normoxic conditions (5% CO_2_).

### MTT Assay

4000 cells per well were seeded into 96-well plate. After 72 h cultivation, MTT (0.5 mg/mL) solution was added into N2a cells of each well, and N2a cells were incubated in 5% CO_2_ at 37 °C for another 4 h. DMSO was then added to dissolve the formazan, and the absorbance of N2a cells at 570 nm was measured using a microplate reader.

### Quantitative Real-Time PCR

Total RNA was extracted using Trizol Regent Kit (RNAiso Plus, Takara, Japan). Then cDNA was synthesized using the PrimeScript RT reagent Kit (Takara, Japan). MicroRNAs were reverse transcribed by miRNA First Strand Synthesis Kit (Clontech, Takara, Japan). Subsequently, quantitative real-time PCR analysis was performed with TB Green real-time-PCR Kit (Takara, Japan) using LightCycler 480 System (Roche, Switzerland). The primers used in this experiment are as follows: Cox-2: forward 5′-TGCCAATAGAACTTCCAATCCG-3′ and reverse 5′-TGGTCGGTTTGATGCTACTG-3′; Fth1: forward 5′-AGGATATAAAGAAACCAGACCGTG-3′ and reverse 5′-TCAGTAGCCAGTTTGTGCAG-3′; Mfn1: forward 5′-GGTGGAAATACAGGGCTACAG-3′ and reverse 5′-ATGCCACCTTCATATGTCTCC-3′.

The expression of mtDNA was detected according to the previous description. Total DNA was isolated by using TIANcombi DNA Lyse&Det PCR Kit (TIANGEN, China). Then, quantitative real-time PCR was performed as above. mtDNA was determined using primers for NADH dehydrogenase subunit 5: forward 5′-ACGAAAATGACCCAGACCTC-3′ and reverse 5′-GAGATGACAAATCCTGCAAAGATG-3′; β-globin: forward 5′-ATGATCCTGTTGCATACCAGTAG-3′ and reverse 5′-TGCCAAAACCCTAGTTGACC-3′. The relative expression ratio of each gene or mtDNA was calculated by the 2^−ΔΔCt^ method.

#### Western Blot

After each treatment, N2a cells were lysed in SDS buffer for 30 min. The protein assay kit (Beyotime Biotech, Shanghai, China) was used to evaluate the total protein concentrations. Cell lysate was separated with 10% SDS-PAGE and then transferred to PVDF membranes. Subsequently, the membranes were blocked and then incubated with primary antibodies overnight at 4 °C, followed by incubation with secondary antibodies for 1 h at room temperature and detection with an ECL kit (Thermo Scientific, USA). The antibodies are as follows: GPx-4 (1:2000, ab125066, Abcam), p22phox (1:1000, ab75941, Abcam), Mfn1 (1:1000, ab221661, Abcam), and GAPDH (1:5000, ab9485, Abcam).

#### Determination of Glutathione/Oxidized Glutathione (GSH/GSSG), GSH, Malondialdehyde (MDA), Superoxide Dismutase (SOD), and Iron Content

The levels of GSH in cell extracts were analyzed using the Glutathione Assay kit (Abcam, cat. no. ab239727). Total glutathione/oxidized glutathione (GSH/GSSG) was determined using the Total GSH/GSSG Assay kit (Abcam, cat. no. ab138881). MDA levels were determined using a Lipid Peroxidation (MDA) Assay kit (Abcam, cat. no. ab118970). The activity of SOD was measured with the Superoxide and Dismutase Assay kit (Solarbio, cat no. BC0170). The content of iron in cells or tissue lysates was detected using the Iron Assay Kit (Abcam, cat. no. ab83366).

#### Measurement of the Mitochondrial Membrane Potential (MMP)

MMP was measured by using JC-1 Assay Kit (cat. no. #1130-5; BioVision). The cells were washed with PBS and then incubated with 10 µg/mL JC-1 for 30 min in the dark. The fuorescence intensity of the JC-1 aggregates/monomers (red fuorescence for JC-1 aggregates, green fuorescence for JC-1 monomers) were taken under a fuorescence microscopy (Leica, Heerbrugg, Germany). Digital images were analyzed with ImageJ software (NIH, Bethesda, MD, USA).

In addition, for cerebral tissue cells in animal experiments, cells were treated with JC-1 staining and then detected using flow cytometry (Beckman Coulter, USA). The ratio between green fluorescence in the B4 quadrant and red fluorescence in the B2 quadrant indicated the level of mitochondrial depolarization.

#### Statistical Analysis

Graphpad Prism 8.0 (GraphPad Software, La Jolla, USA) was used to statistically analyze all graphs in this article. Measurement data were presented as mean ± standard deviation (SD). Differences between groups were compared by unpaired Student’s *t* test or one-way analysis of variance (ANOVA). *p* < 0.05 was indicated a statistical significance.

## Results

### Selenium Treatment Alleviates Cerebral I/R Injury and Oxidative Stress in Mice

**Fig. 1:**
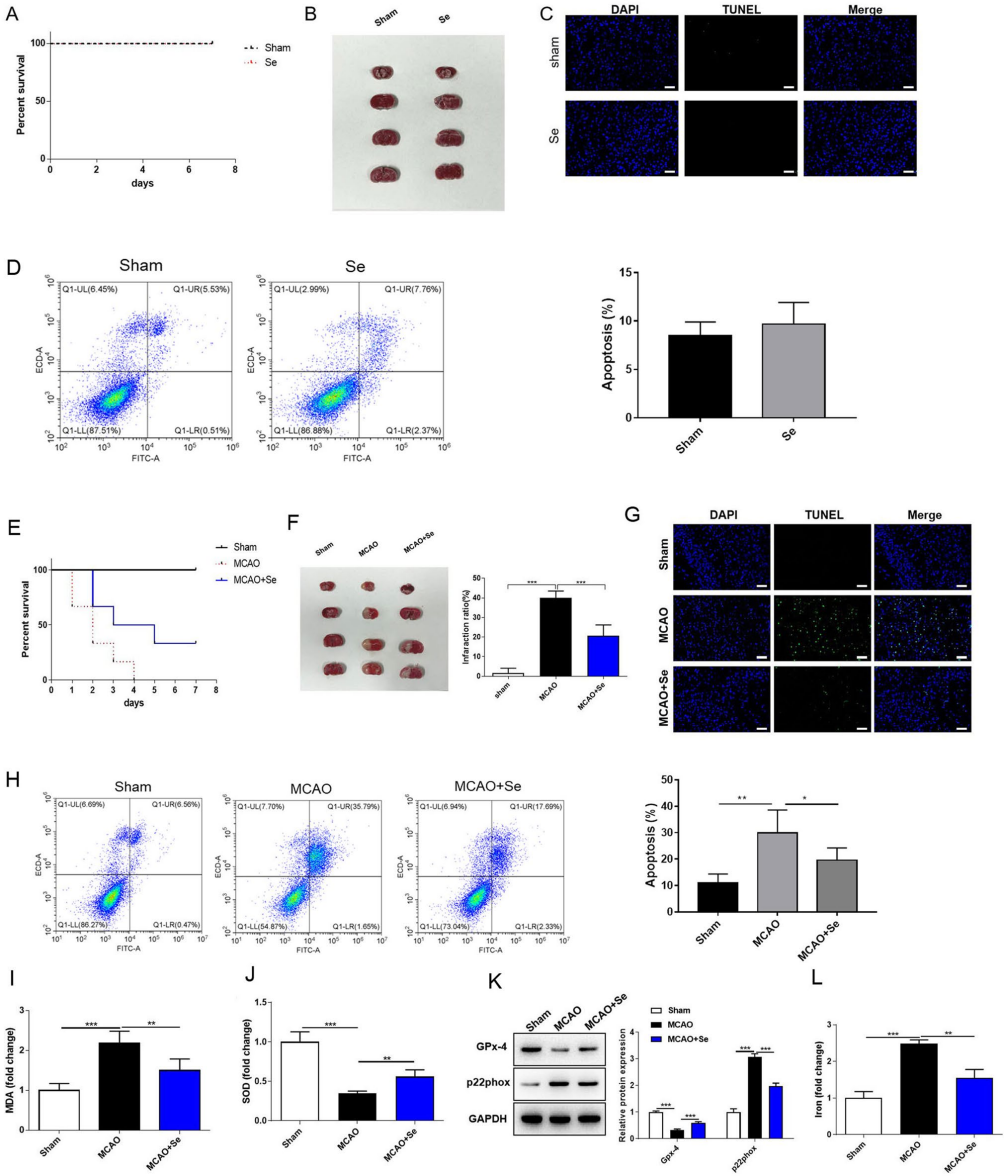
Se treatment alleviates cerebral I/R injury and oxidative stress in mice. We first established MCAO model in mice, the group of which was named as: Sham, MCAO, and MCAO + Se. Then the survival rate of mice was recorded. The infarct area volume and apoptosis level of brain tissues was observed. Moreover, the levels of oxidative stress related indicators were detected. **A** and **E** The survival rate of mice in each group was recorded. **B** and **F** TTC staining showed the infarct area of mouse brain tissues. **C** and **G** The apoptosis of mouse brain tissue in TUNEL staining. Scale bar = 200 μm. **D** and **H** The apoptosis of mouse brain tissues detected by flow cytometry. **I**–**J** The level of MDA and SOD was detected using corresponding kit. **K** The protein level of GPx-4 and p22phox was detected by western blot. **L** The content of iron was detected using corresponding kit. Data are shown as mean ± SD. *, *p* < 0.05; **, *p* < 0.01; ***, *p* < 0.001

Our previous results showed that compared with the sham group, Se treatment alone (intraperitoneal injection of 0.5 mg/kg/day of selenite sodium) had no significant toxic effect on mice, including the survival rate (Fig. 1A), cerebral infarction area (Fig. 1B) and brain cell apoptosis (Fig. 1C, D). Thus, the dose of Se treatment was applied for the established MCAO model mice to explore the therapeutic effect of Se on cerebral I/R injury, the groups of which were as follows: Sham, MCAO, and MCAO + Se treatment group. The result showed that Se treatment significantly improved the survival rate of mice compared with the MCAO group (Fig. 1E). In addition, there was a serious cerebral infarction of mouse brain tissue in MCAO group, indicating that the cerebral I/R modeling was successful. Compared with MCAO group, the infarct area was effectively reduced after Se treatment (Fig. 1F). Cerebral I/R injury exerted an increase of TUNEL fluorescence in MCAO group, indicating that apoptosis of brain cells in the affected area was increased. After Se treatment, however, a decreased TUNEL intensity was observed (Fig. 1G), and reduced cell apoptosis rate detected by flow cytometry assay (Fig. 1H). Subsequently, we detected the level of oxidative biomarker MDA and antioxidant marker SOD with the relevant detection kits. Compared with sham operation, the MDA level in MCAO group was apparently higher and the SOD level was markedly lower. While Se treatment notably reverse the effect of MCAO group on the level of MDA and SOD (Fig. 1I, J). Oxidative stress usually leads to the increase of p22phox protein expression and the decrease of GPx-4. At the same time, western blot showed that the protein expression of GPx-4 in MCAO group was significantly suppressed, yet p22phox was induced. Se treatment significantly reversed the changes caused by I/R injury (Fig. 1K). Finally, by detecting the content of iron in brain tissue, we found that the content of iron in MCAO model group increased significantly, and Se treatment could apparently reverse the content of iron (Fig. 1L). These data revealed that Se treatment could lessen oxidative stress in mouse cerebral I/R injury.

### Selenium Treatment Attenuates Oxidative Stress by Alleviating Ferroptosis

**Fig. 2:**
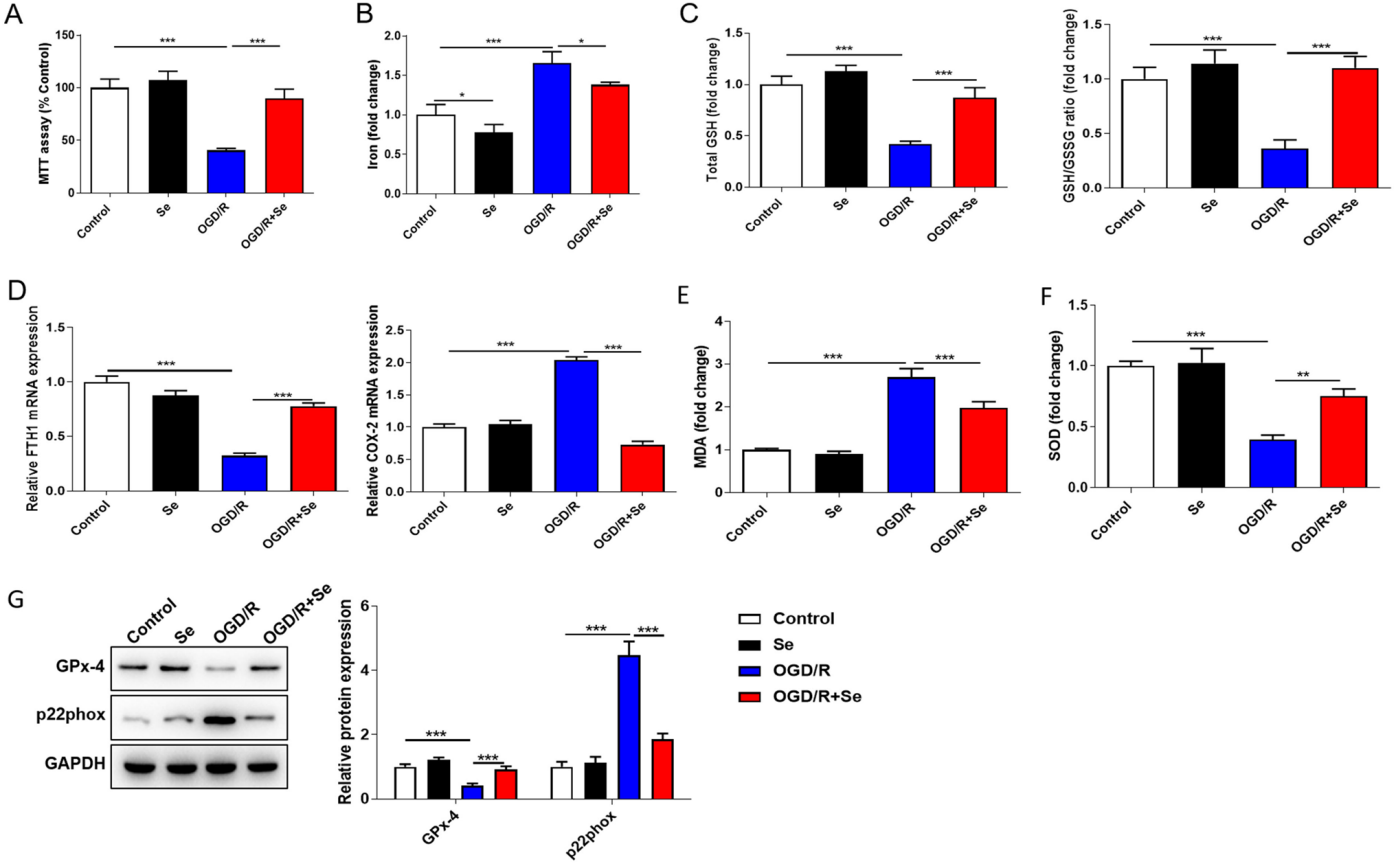
Se treatment attenuates oxidative stress by alleviating ferroptosis. N2a cells were used to construct OGD/R model, and then the cells were treated with Se. The groups are as follows: Control, Se, OGD/R, and OGD/R + Se. Then, the cell viability, oxidative stress indicators and ferroptosis-related factors were analyzed. **A** MTT assay was performed to detect the cell viability in N2a cells of each group. **B** The content of iron in N2a cells was detected using corresponding kit. **C** The levels of GSH and GSH/GSSG in N2a cells were measured using corresponding kits. **D** QRT-PCR was used to detect the expression of Cox-2 and Fth1 in N2a cells. **E**–**F** The level of MDA and SOD was detected using corresponding kit. **G** The protein level of GPx-4 and p22phox was detected by western blot. Data are shown as mean ± SD (n = 3). *p < 0.05; **p < 0.01; ***p < 0.001

Previous studies have shown that oxidative stress can induce ferroptosis and mitochondrial dysfunction in neural cells [17]. Through in vivo experiments, we found that Se treatment reduced cerebral I/R injury and affected the content of iron in brain tissue. Thus, we want to explore whether Se affected ferroptosis and mitochondrial function in neural cells. The OGD/R model was constructed by N2a cells, and then the cells were treated with Se. MTT assay showed that the cell viability was significantly inhibited in OGD/R group, but increased significantly after Se treatment (Fig. 2A). Iron assay kit was used to detect the content of iron in cells. From the results, we observed that the content of iron was increased significantly in OGD/R group, and Se treatment could reduce the content of iron (Fig. 2B). The levels of GSH and GSH/GSSG in N2a cells were measured with corresponding kit, both of which were apparently repressed with OGD/R treatment, while elevated in OGD/R + Se treatment group (Fig. 2C). As ferroptosis related factors, Cox-2 and Fth1 are differentially expressed under the process of cell ferroptosis. QRT-PCR assay showed the expression of Fth1 depressed and Cox-2 increased obviously in OGD/R group, but the above changes were significantly reversed in OGD/R + Se group (Fig. 2D). The level of MDA was induced and SOD was suppressed by OGD/R treatment, both of which were fell back in Se + OGD/R treatment group (Fig. 2E–F). The protein expression of GPx-4 was obviously suppressed but p22phox was induced in OGD/R group. However, OGD/R + Se treatment markedly reversed the changes caused by OGD/R treatment alone (Fig. 2G). These results indicated that Se treatment could attenuate oxidative stress through alleviating ferroptosis induced by OGD/R.

### Selenium Treatment Up-Regulates Mfn1 Expression and Promotes Mitochondrial Fusion

**Fig. 3:**
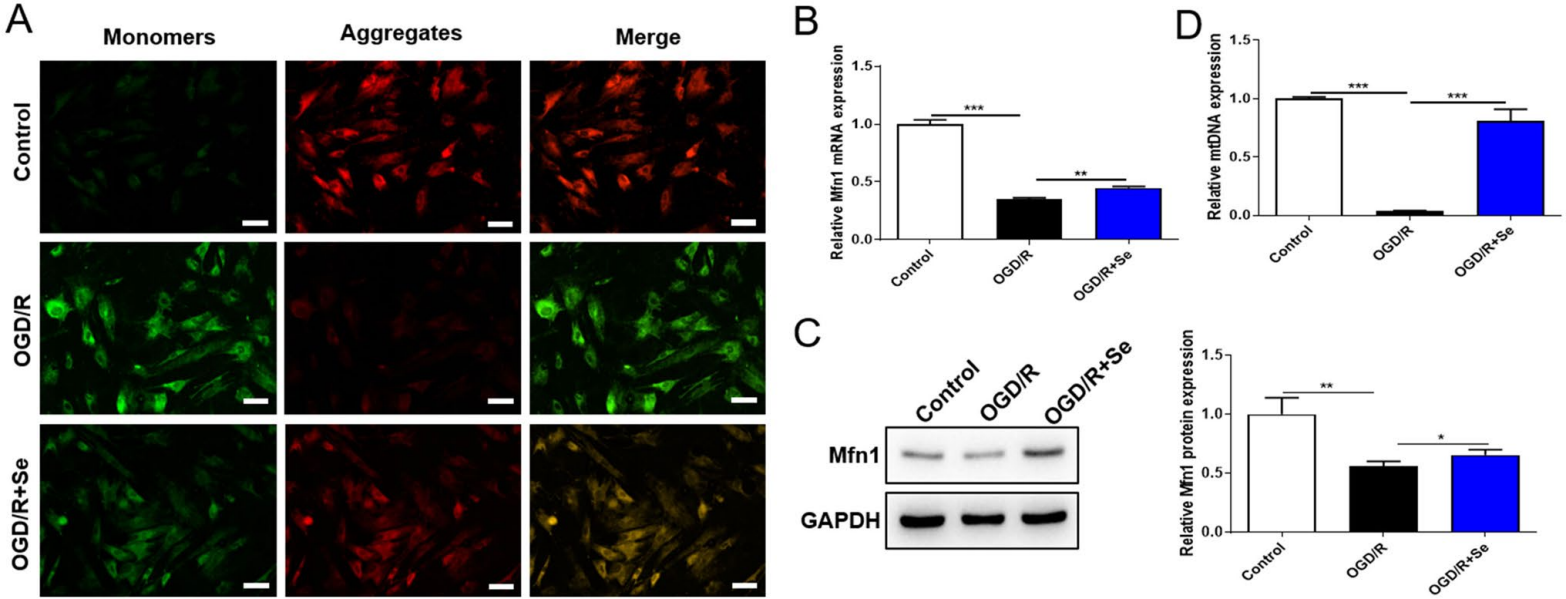
Se treatment up-regulates Mfn1 expression and promotes mitochondrial fusion. Firstly, OGD/R mode was established with N2a cells, and then treated with Se. The cells were divide into three groups: Control, OGD/R, OGD/R + Se. The MMP was detected in N2a cells. Then the expression of Mfn1 in N2a cells in different groups was analyzed. Furthermore, the level of mtDNA was analyzed. **A** JC-1 staining was performed to observe MMP in N2a cells of each group. Scale bar = 5 μm. **B** and **C** The mRNA and protein expression of Mfn1 in N2a cells of each treatment group. **D** The level of mtDNA was analyzed by qRT-PCR. Data are shown as mean ± SD (n = 3). *p < 0.05, **p < 0.01, ***p < 0.001

To observe whether Se treatment mediated the function of mitochondrial membrane, we first detected the changes of MMP through JC-1 staining. The result revealed that Se treatment reversed the decrease of MMP triggered by OGD/R treatment (Fig. 3A). Studies have shown that Mfn1 is crucial for mitochondrial morphology, especially mitochondrial fusion [20]. Therefore, we then detected the gene and protein expression of Mfn1 in N2a cells of each treatment. From the results, OGD/R treatment inhibited the expression of Mfn1, and OGD/R + Se treatment apparently induced the expression of Mfn1 (Fig. 3B, C). In addition, the content of mtDNA in OGD/R group was notably decreased, while increased significantly after Se treatment (Fig. 3D). These observations verified that Se might promote the process of mitochondrial fusion to maintain normal mitochondrial biological function by inducing Mfn1 expression.

### Mfn1 Alleviates Ferroptosis by Promoting Mitochondrial Fusion

**Fig. 4:**
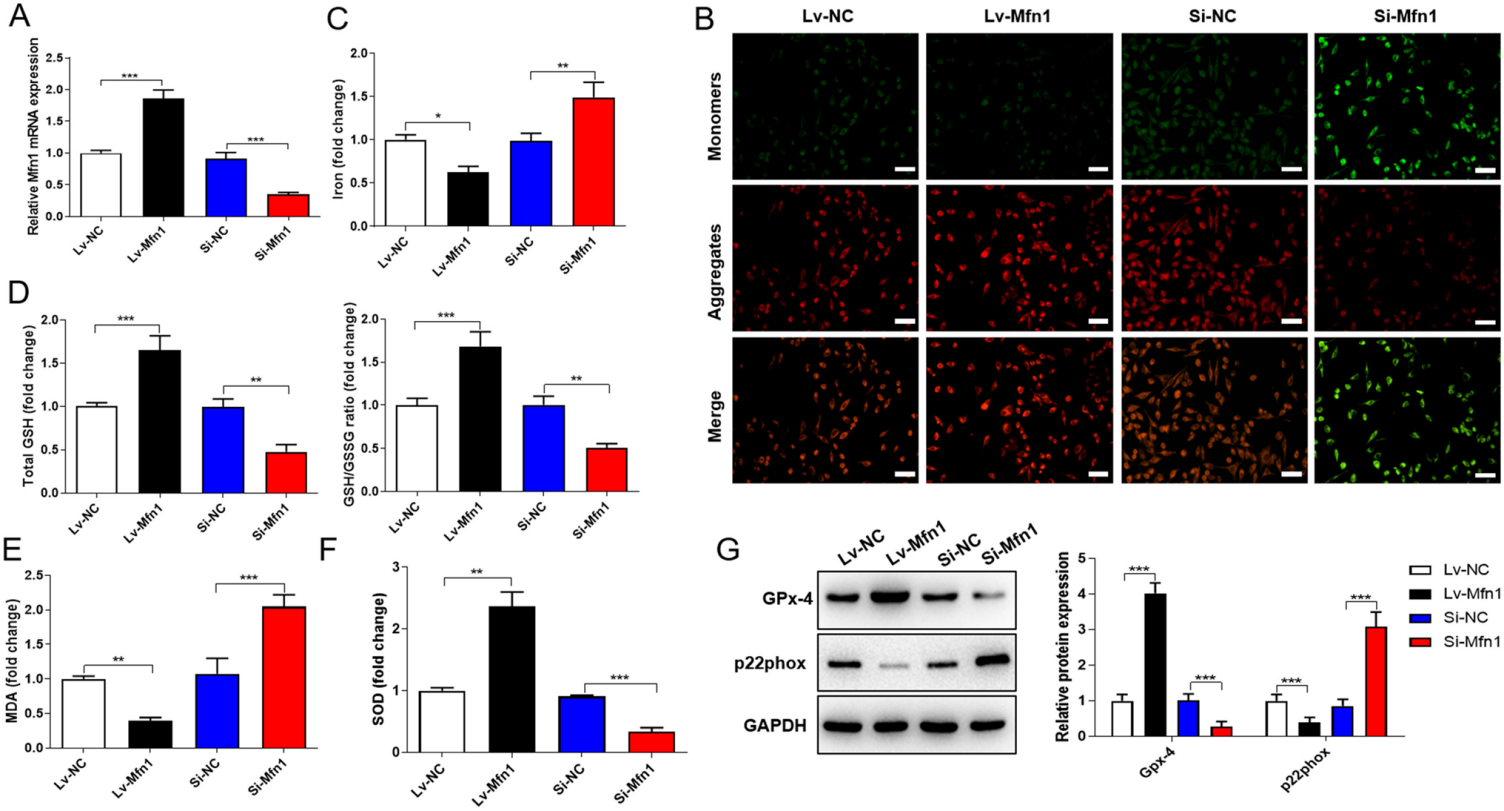
Mfn1 alleviates oxidative stress and ferroptosis by promoting mitochondrial fusion. After OGD/R, N2a cells were transfected with differentially expressed Mfn1, and the groups are as follows: Lv-Mfn1, Lv-NC, Si-Mfn1, and Si-NC. Then the indicators of oxidative stress and ferroptosis related factors of N2a cells in different groups were analyzed. **A** The expression of Mfn1 was detected by qRT-PCR. **B** JC-1 staining was performed to detect the MMP in N2a cells of each group. Scale bar = 100 μm. **C** The iron content in N2a cells of each group. **D**–**F** The levels of GSH, GSH/GSSG, MDA and SOD were measured using corresponding kits. **G** The protein level of GPx-4 and p22phox in N2a cells was detected by western blot. Data are shown as mean ± SD (n = 3). *p < 0.05; **p < 0.01; ***p < 0.001

In order to further confirm that Mfn1 was involved in the regulation of cerebral I/R injury, we overexpressed or knocked down Mfn1 in N2a cells, and then the cells were treated with OGD/R. The transfection efficiency was confirmed by qRT-PCR to exclude the effect of off-target (Fig. 4A). Through JC-1 staining, we observed that MMP was apparently increased after overexpression of Mfn1 and decreased in Mfn1 knockdown group (Fig. 4B). Subsequently, we found that the content of iron was negatively correlated with expression of Mfn1 (Fig. 4C). And the levels of GSH and GSH/GSSG was positively with expression of Mfn1 (Fig. 4D). In addition, overexpression of Mfn1 significantly reduced MDA and p22phox expression but induced SOD and GPx-4, while knockdown of Mfn1 showed an opposite trend (Fig. 4E–G). Taken together, Mfn1 might emerge the ability to regulate cellular ferroptosis and anti-oxidative stress caused by OGD/R injury through promoting mitochondrial fusion.

### Selenium Treatment Alleviates Cerebral I/R Injury by Regulating Mfn1 In Vivo

**Fig. 5:**
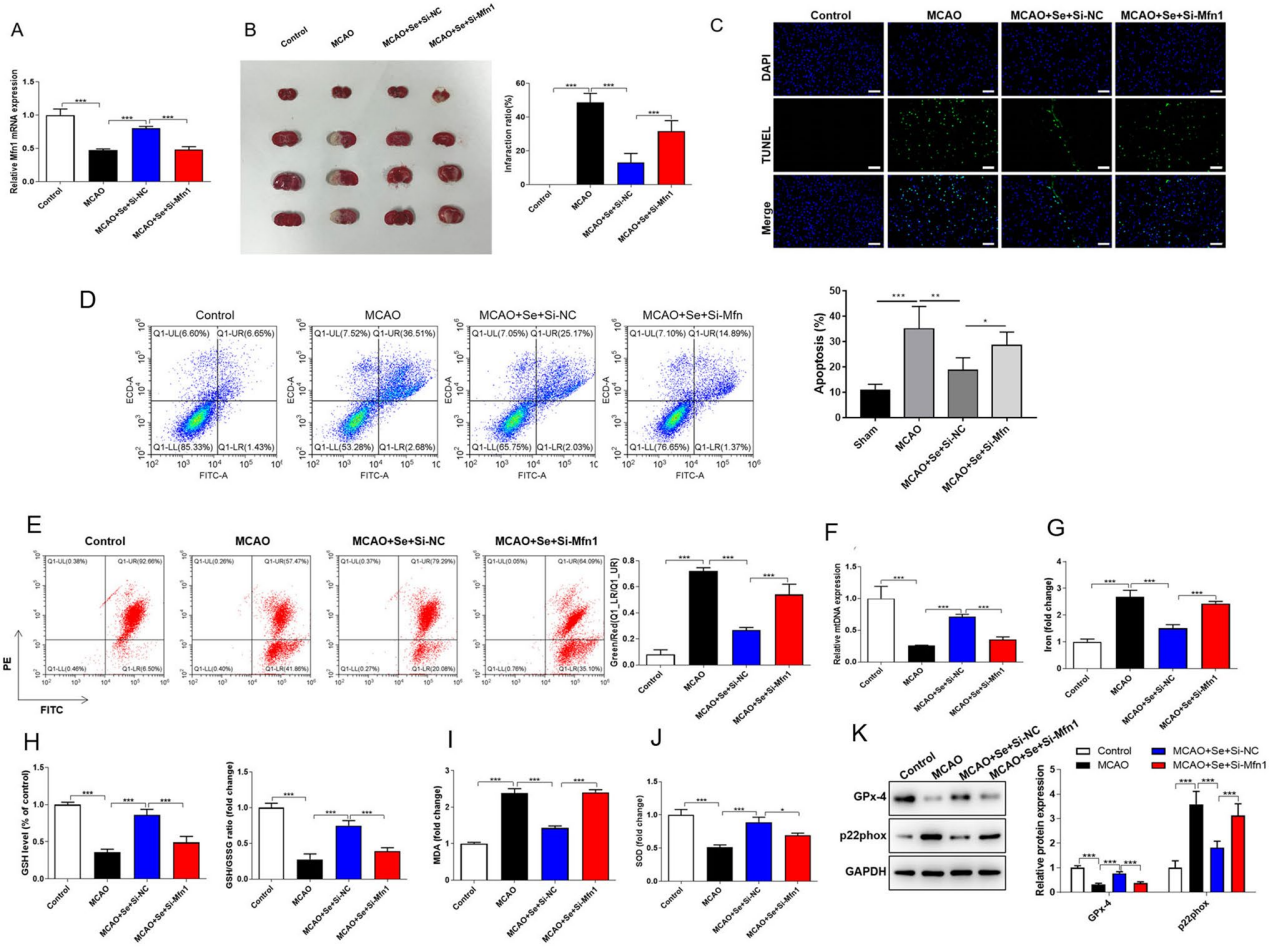
Molecular mechanism Se treatment alleviates cerebral I/R injury in vivo. The group name of MCAO model mice was: Sham, MCAO, MCAO + Se + Si-NC, and MCAO + Se + Si-Mfn1. The expression of Mfn1 was analyzed in mice. Then the infarct area volume and apoptosis level of mice brain tissues was observed. Moreover, the levels of oxidative stress and ferroptosis related indicators were detected. **A** The expression of Mfn1 was detected by qRT-PCR. **B** The infarct area of mice brain tissues in TTC staining. **C** The apoptosis of mouse brain tissues in TUNEL staining. Scale bar = 200 μm. **D** The apoptosis of mouse brain tissues was detected by flow cytometry. **E** JC-1 staining was performed to detect the MMP in mice brain tissues. **F** The level of mtDNA was analyzed by qRT-PCR in mice brain tissues. **G** The iron content in mouse brain tissues. **H** The levels of GSH, GSH/GSSG and **I** MDA and **J** SOD was detect using corresponding kit. **K** The protein level of GPx-4 and p22phox was detected by western blot in N2a cells. Data are shown as mean ± SD. *p < 0.05; **p < 0.01; ***p < 0.001

To extend the effect of Se treatment in vivo situation, mice were treated and grouped as follows: control, MACO, MCAO + Se + Si-NC, and MCAO + Se + Si-Mfn1. QRT-PCR result showed that the expression of Mfn1 was notably decreased after MCAO treatment, but Mfn1 was reversed by MCAO + Se + Si-NC group and then decreased again in MCAO + Se + Si-Mfn1 group (Fig. 5A). TTC staining showed that Se treatment significantly reduced the cerebral infarction area of MCAO mice, which was increased after knockdown of Mfn1 (Fig. 5B). Besides, TUNEL assay showed that the level of apoptosis decreased significantly after Se treatment, but it was increased under the condition of Mfn1 knockdown (Fig. 5C). The similar results were observed after performing flow cytometry apoptosis assay (Fig. 5D). Se treatment promoted the increase of MMP and mtDNA level in MCAO mice, while knocking down Mfn1 neutralized the effects (Fig. 5E, F). Furthermore, subsequent experiments were carried out to detect the indexes-related to oxidative stress and ferroptosis in the brain of mice, which were consistent with the trends in vitro experiments. Se treatment significantly inhibited the levels of oxidative stress and ferroptosis in MACO mice, while knockdown of Mfn1 showed an opposite trend (Fig. 5G–K). The above results demonstrated Se alleviated cerebral I/R injury to inhibit ferroptosis and reduce oxidative stress by up-regulating Mfn1.

## Discussion

In the past decades, Se and its compounds have attracted much attention because of their multiple biological functions and unique mechanisms of incorporation into proteins. The researchers first found a specific role of Se in the brain in children with intractable epilepsy. These children had low glutathione peroxidase activity and their clinical symptoms were improved after Se supplementation [21]. Previous studies have shown that elemental Se can regulate some cellular biochemical processes, mainly including mitochondrial function, oxidative stress, and apoptosis [22–24].

In this study, we investigated the neuroprotective effects of Se in vivo and in vitro. By constructing a MCAO animal model, we confirmed the therapeutic effect of Se on cerebral I/R injury. It is known that Se homeostasis and antioxidant selenoproteins in brain are of great significance in brain diseases [25, 26]. Micronutrient Se is often referred to as "antioxidant" in mineral supplements, which is enough to explain its achievements in antioxidation. As a component of enzymes and other proteins in biological antioxidant system, Se plays a beneficial role in catalyzing redox reaction and maintaining redox homeostasis [27–29]. Subsequently, through the OGD/R model, we found that Se could significantly inhibit the oxidative stress caused by OGD/R injury in vitro.

A characteristic of cell ferroptosis is the accumulation of lipid reactive oxygen species, leading to oxidative stress and cell death, which has been shown to have significant implications in diverse brain diseases [30, 31], such as Alzheimer’s disease [32], Parkinson’s disease [33], and cerebral ischemic disease [34]. Ferroptosis process is closely related to oxidative stress, and usually accompanied by the increase of iron content. Interestingly, we found that Se treatment significantly reversed the increase of iron content in mice induced by MCAO, suggesting that Se might play a protective role by inhibiting ferroptosis.

A recent study using cardiac MRI suggested that I/R injury could cause local accumulation of iron from hemoglobin and heme release at the focal lesion [35], resulting in excessive reactive oxygen species and pathological events such as inflammation [36]. There was evidence that I/R injury related neuronal damage could be rescued by ferroptosis inhibitors, such as liprostatin-1 and ferrostatin-1, authenticating ferroptosis is a direct contributor to brain I/R injury [37]. Therefore, we deeply explored whether Se treatment could reduce the damage of N2a cells after ODG/R treatment by affecting the level of ferroptosis. Our study found that Se played a neuroprotective role by reducing ferroptosis and oxidative stress of N2a cells caused by OGD/R.

When the autophagy-lysosomal pathway, especially mitophagy, is blocked, the damaged mitochondria will accumulate excessively rather than degrade normally, resulting in the loss or injury of brain neurons, which becomes an important inducement of brain I/R injury [38]. Evidence has suggested that Se can protect the structural integrity of mitochondria by activating mitochondrial biogenic signals in hyperglycemic rats, so as to improve the cerebral I/R injury [39]. In our study, Se treatment resulted in an up-regulation expression of Mfn1, thus promoting mitochondrial fusion of N2a cells in the OGD/R model. Also, animal experiments also showed that Se alleviated cerebral I/R injury to inhibit ferroptosis and reduce oxidative stress by up-regulating Mfn1.

In conclusion, our study proves that Se can alleviate the damage of cerebral I/R injury, and its neuroprotective effects might be related to cellular oxidative stress, mitochondrial fusion and ferroptosis level. Complementary to previous studies, these findings provide a theoretical basis for clinical diagnosis and treatment of cerebral I/R injury.

## Data Availability

The data used to support the findings of this study are available from the corresponding author upon request.

## Abbreviations

Se: Selenium
MCAO: Middle cerebral artery occlusion
OGD/R: Oxygen–glucose deprivation and reoxygenation
MDA: Malondialdehyde
SOD: Superoxide dismutase
GSH: Glutathione
GSH/GSSG: Glutathione/oxidized glutathione
GPX: Glutathione peroxidase
COX-2: Cyclooxygenase-2
FTH1: Ferritin heavy chain 1
TTC: 2,3,5-Triphenyltetrazolium chloride
